# Exogenous testosterone exacerbates pre-neoplastic lesions in the prostate of NKX3.1-deficient mice

**DOI:** 10.1186/s42826-026-00284-8

**Published:** 2026-06-26

**Authors:** Su Jeong Lim, Jin Ju Park, Ji Eun Kim, Hee Jin Song, Ayun Seol, Su Ha Wang, Ye Eun Ryu, Ye Ryeong Kim, Jun Go, Dae Youn Hwang

**Affiliations:** 1 Department of Biomaterials Science (BK21 FOUR Program)/Life and Industry Convergence Research Institute/Laboratory Animal Resources Center, College of Natural Resources & Life Science, Pusan National University, Miryang, 50463 Republic of Korea; 2 Department of Biology and Chemistry, Changwon National University, Changwon, 51140 Republic of Korea; 3 Global Institute for Advanced Nanoscience & Technology (GIANT), Changwon National University, Changwon, 51140 Republic of Korea

**Keywords:** NKX3.1, Testosterone, Pre-neoplastic lesions, Prostatic hyperplasia

## Abstract

**Background:**

NKX3.1 is a prostate-specific tumor suppressor that is frequently downregulated during the early stages of prostate cancer. Although NKX3.1 knockout (KO) mice develop spontaneous epithelial abnormalities, these lesions rarely progress beyond early neoplastic changes without additional oncogenic stimulus. Therefore, we investigated whether exogenous testosterone (TS) exacerbates early-stage, pre-neoplastic lesions in the prostate of NKX3.1 KO mice. Alterations in prostate weights of male reproductive organs (testis, seminal vesicles, and prostate lobes), histopathological lesion scores, apoptotic proteins, and angiogenic proteins were analyzed in C57BL/6 NKX3.1^em1Hlee^/Korl KO (NKX3.1 KO) mice injected with TS for six weeks.

**Results:**

The weight of testis, seminal vesicles and ventral prostate was commonly changed in TS-treated mice of wild type (WT) and NKX3.1 KO group, while those of the dorsolateral and anterior prostate were only increased in TS-treated NKX3.1 KO group compared to those of WT. Histopathological lesion severity was greater in TS-treated NKX3.1 KO mice, with the highest lesion scores observed in the high-dose TS (HiTS)-treated KO group, and a similar pattern was observed for p53 staining. The expression levels of apoptotic and angiogenic proteins were significantly increased in TS-treated NKX3.1 KO mice compared to the same group of WT mice.

**Conclusions:**

These findings suggest that the exogenous TS exacerbates early-stage, pre-neoplastic lesions in the prostate of NKX3.1 KO mice, consistent with a gene–hormone synergistic interaction. The mechanistic basis of this synergy remains to be defined.

**Supplementary information:**

The online version contains supplementary material available at 10.1186/s42826-026-00284-8.

## Background

Prostate cancer is one of the most frequently diagnosed malignancies and a leading cause of cancer-related mortality among men worldwide [1]. Its initiation and development are driven by a complex interplay of hormonal regulation, genetic alterations, and environmental factors. Androgen signaling, particularly through testosterone (TS) and its more potent derivative dihydrotestosterone (DHT), plays a pivotal role in maintaining the growth, differentiation, and function of the prostate gland [2–4]. While physiological levels of TS are required for normal prostate homeostasis, the excessive or dysregulated TS exposure has been associated with abnormal cellular proliferation, prostatic hyperplasia, and may influence prostate cancer risk in both clinical and experimental contexts [5–7].

Among genetic factors contributing to prostate cancer susceptibility, NKX3.1, a prostate-specific homeobox gene located at chromosome 8p21, has been extensively characterized as an early-onset tumor suppressor [8]. This gene is highly expressed in normal prostate epithelial cells and plays a crucial role in regulating epithelial cell differentiation [9], suppressing oxidative stress [10], and maintaining DNA integrity [11]. Notably, loss or reduction of NKX3.1 expression is one of the earliest molecular events observed in human prostate carcinogenesis and is detected in over 60–80% of early-stage prostate cancers [8, 12]. Mouse models lacking NKX3.1 (NKX3.1 knockout, or KO) recapitulate many of the histopathological features of human prostatic intraepithelial neoplasia, including epithelial hyperplasia, nuclear atypia, and increased proliferation [13]. However, these mice do not spontaneously develop invasive prostate cancer, suggesting that NKX3.1 loss alone is insufficient for full malignant transformation but may act as a “gatekeeper” that predisposes the prostate to secondary oncogenic insults [9, 13, 14].

Meanwhile, the previous studies have demonstrated that androgen injection into rodents can induce the prostatic epithelial proliferation and even lead to the neoplastic changes under certain conditions [15, 16]. Moreover, hormone replacement therapy in aging men—while restoring systemic TS levels—has raised questions regarding its safety in individuals at high risk of prostate cancer [17]. However, despite extensive research on NKX3.1 KO mice [9, 13, 18], no previous studies have directly examined the injection effects of exogenous TS on the development of early-stage, pre-neoplastic lesions in NKX3.1-deficient mice. Understanding the combinatorial effects of genetic vulnerability and androgenic stimulation is thus essential for elucidating the early events of prostate cancer development and for identifying potential windows for therapeutic intervention, particularly given the clinical relevance to TS replacement therapy safety [19, 20].

In this study, we investigated whether the injection of exogenous TS exacerbates early-stage, pre-neoplastic lesions during NKX3.1 deficiency. We hypothesized that NKX3.1 loss creates a permissive genetic background that sensitizes the prostate epithelium to androgen-dependent early neoplastic remodeling. This approach offers a biologically grounded in vivo context to explore gene–hormone synergy at the initiation stage and may help contextualize potential risks relevant to testosterone replacement therapy (TRT) in genetically susceptible individuals.

## Methods

### Animals

C57BL/6N-NKX3.1^em1Hlee^ KO mice, generated by CRISPR/Cas9-mediated genome editing, were obtained from the Laboratory Animal Resources Bank (LAREB), Department of Laboratory Animal Resources, National Institute of Food and Drug Safety Evaluation (NIFDS, Cheongju, Korea) [21]. Wild-type (WT) and NKX3.1 KO mice were maintained at the Laboratory Animal Resources Center of Pusan National University, which is accredited by the Korea Food and Drug Administration (KFDA; Unit No. 000231) and the Association for Assessment and Accreditation of Laboratory Animal Care International (AAALAC; Unit No. 001525). Mice were housed under a 12 h light/12 h dark cycle (lights on at 07:00 and off at 19:00) at a controlled temperature of 22 ± 2°C and relative humidity of 50 ± 10% in individually ventilated cages (IVCs; 17 × 32 × 14 cm) within an ENVIRO-GARD™-B Environmental Control System (Lab Products Inc., Seaford, DE, USA). Shelter/Igloo enrichment was provided. Mice had *ad libitum* access to distilled water and a standard chow diet (Samtako BioKorea Co., Osan, Korea). Animal protocol was approved by the Institutional Animal Care and Use Committee of Pusan National University (PNU-IACUC; Approval No. PNU-2017–1719).

### Testosterone injection

**Fig. 1:**
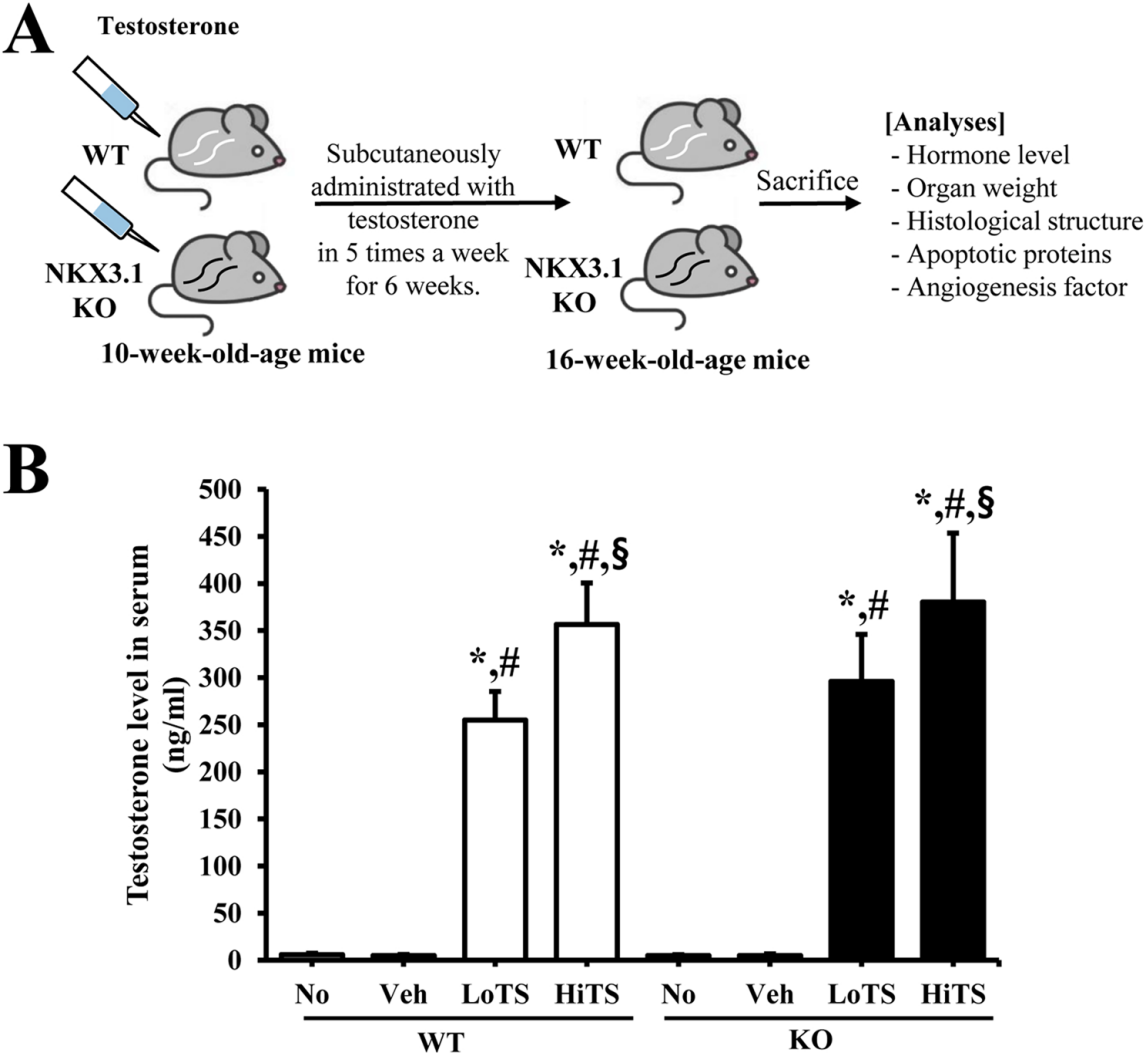
Experimental scheme and serum testosterone concentration following TS treatment. (**A**) Experimental scheme for TS injection and analysis. WT and NKX3.1 KO mice were subcutaneously injected by LoTS or HiTS five times per week for six weeks. (**B**) Serum TS level. After collecting serum, their levels were measured by ELISA at the end of the treatment period. Data are presented as mean ± SD. *, *p* < 0.05 vs. No; ^#^, *p* < 0.05 vs. Veh; ^§^, *p* < 0.05 vs. LoTS. Statistical significance was determined by ordinary one-way ANOVA followed by Tukey’s multiple-comparisons test. Abbreviations: wild type, WT; knockout, KO; Vehicle, Veh; low-dose testosterone, LoTS; high-dose testosterone, HiTS

To inject TS into WT and NKX3.1 KO mice, 10-week-old mice (male) were divided into four treatment groups each (*n* = 5 per group): no treatment (No), vehicle (Veh) (Olive oil, Sigma-Aldrich, St. Louis, MO, USA), low-dose testosterone (LoTS), and high-dose testosterone (HiTS)-treated groups. TS propionate (Tokyo Chemical Ins. Co., Tokyo, Japan) was dissolved in olive oil and subcutaneously injected five times per week for six consecutive weeks. The TS doses (3 and 6 mg/kg) were selected based on published mice androgen-injection protocols within a comparable mg/kg range. TS injection at 3 mg/kg has been used to induce TS-responsive prostate epithelial hyperplasia phenotypes in mice [22], and higher-dose TS propionate (e.g., 7.5 mg/kg, s.c.) has also been used in mice studies to elicit robust androgen-driven prostate growth phenotypes [23]. Accordingly, we designated 3 mg/kg as a LoTS-treated group and 6 mg/kg as a HiTS -treated group to evaluate dose-dependence of early-stage lesion phenotypes in NKX3.1-deficient prostates. LoTS and HiTS corresponded to 3 mg/kg and 6 mg/kg, respectively, delivered in a volume of 100 μL per injection. The mice in the Veh-treated group were received the same volume of olive oil following the same injection schedule. Body weight was measured once weekly throughout the treatment period. On the day following the final TS injection, mice were euthanized using CO₂ inhalation. Whole blood was collected from the abdominal aorta, and the testis, seminal vesicles (SV), ventral prostate (VP), dorsolateral prostate (DLP), and anterior prostate (AP) were excised from the abdominal cavity of mice, and organ weights were recorded immediately after collection. Each region of the prostate tissue was used for histological and molecular analyses (Fig. 1A).

### ELISA

The concentration of TS in serum was measured using a TS ELISA kit (Cayman, Ann Arbor, MI, USA), following the manufacturer’s instructions. Briefly, whole blood was collected using BD Microtainer® SST™ Tubes (BD Biosciences, Franklin Lakes, NJ, USA) and centrifuged at 1,500 × g for 15 minutes at 4°C to obtain serum. A total of 50 μL of serum was then added to a 96-well microplate pre-coated with anti-testosterone antibodies. After incubation with enzyme-conjugated reagents and substrate, the absorbance of mixture was measured at 450 nm using a VersaMax plate reader (SoftMax® Pro software, v5.2; Molecular Devices, Sunnyvale, CA, USA). TS concentrations were determined by comparing the absorbance values to a standard curve of TS.

### Histological analysis

DLP, VP, and AP of prostate were fixed in 4% formaldehyde for 7 days. These tissues were processed, embedded in paraffin, and sectioned at 4 μm thickness for histological analysis. And then, they were deparaffinized, rehydrated through a graded ethanol series, and stained with hematoxylin and eosin (H&E, Sigma-Aldrich) to evaluate histological structure and epithelial morphology. After optical microscopic examination of the prostate tissue sections at 400 x magnification, lesion type (hyperplasia/adenoma) and histopathological features were characterized by Professor Beum Seok Han at Hoseo University (Asan, Korea). In addition, histopathological alterations in each prostate lobe were scored in a blinded manner using a 0–4 lesion severity scale, where 0 = normal, 1 = minimal hyperplasia, 2 = slight hyperplasia, 3 = moderate hyperplasia, and 4 = adenoma.

For detection of p53 expression, immunofluorescence staining was performed on additional sections. After antigen retrieval using citrate buffer (pH = 6.0), sections were blocked with 5% normal goat serum at room temperature for 30 minutes and incubated overnight at 4°C with an anti-p53 primary antibody (Thermo Fisher Scientific, Waltham, MA, USA). The sections were then incubated with an Alexa Fluor 488-conjugated secondary antibody (Thermo Fisher Scientific). Finally, the green fluorescence signal for the localization and expression levels of p53 protein were analyzed the stained section using a fluorescence microscope (Moticam pro 285A, Motic Microscopes, Hong Kong, China). For quantitative analysis, p53 immunofluorescence intensity was measured in the epithelial compartment using ImageJ (National Institutes of Health, Bethesda, MD, USA). Corrected total cell fluorescence (CTCF) was calculated as Integrated Density − (ROI area × mean background fluorescence), and values were expressed as fold change normalized to the WT group.

### Western blot

The expression levels of apoptotic and angiogenic proteins were detected as described in a previous study [24]. Briefly, the prostate tissues were homogenized in Pro-prep Protein Extraction Solution (iNtRON Biotechnology Inc., Seongnam, Korea) supplemented with half tablet of a protein inhibitor cocktail (Roche Diagnostics, Mannheim, Germany). Total tissue lysates were centrifuged at 13,000 × g for 5 minutes at 4°C to remove cellular debris, and the supernatants were collected. After determining the protein concentration using a BCA protein assay kit (Thermo Fisher Scientific), the equal amounts of protein (30 μg) were separated by SDS-PAGE on 10% polyacrylamide gels and transferred onto nitrocellulose membrane (Amersham Biosciences, Little Chalfont, UK) for 2 hrs at 40 V. The membranes were blocked with 5% skim milk in Tris-buffered saline containing 0.1% Tween-20 (Thermo Fisher Scientific) (TBST) for 1 hr at room temperature, followed by overnight incubation at 4°C with primary antibodies against vascular endothelial growth factor (PeproTech, Rocky Hill, NJ, USA), protein kinase B (AKT, Cell Signaling Technology, Danvers, MA, USA), phosphorylated AKT (p-AKT, Cell Signaling Technology), and β-actin (1:1,000, Sigma–Aldrich). After washing with TBST, the membranes were incubated with goat anti-rabbit immunoglobulin G (IgG) conjugated with horseradish peroxidase (HRP) (TransGen Biotech Co., Ltd., Beijing, China) for 1 hr at room temperature. Immunoreactive bands were visualized using enhanced chemiluminescence reagents (ECL, Amersham Bioscience) and detected with a FluorChem® FC2 Imaging system (Alpha Innotech Corporation, San Leandro, CA, USA).

### Statistical analysis

Statistical analysis was performed using GraphPad Prism (version 8.0.2; GraphPad Software, Boston, MA, USA). For continuous variables, homogeneity of variance was assessed using the Brown-Forsythe test. When the equal-variance assumption was satisfied, ordinary one-way ANOVA followed by Tukey’s multiple-comparisons test was used. When the assumption was violated, Welch’s ANOVA followed by Games-Howell’s multiple-comparisons test was applied. Histopathological lesion scores were analyzed using the Kruskal–Wallis test followed by Dunn’s multiple-comparisons test. Data are presented as mean ± SD, and differences were considered statistically significant at *p* < 0.05.

## Results

### Increase in serum testosterone levels after testosterone injection in NKX3.1 KO mice

Firstly, we confirmed that TS injection increased serum TS levels in both WT and NKX3.1 KO mice. To achieve this, the serum TS concentrations were determined in the serum of WT and NKX3.1 KO mice after subcutaneously injection for 6 weeks. Weekly body weight monitoring during the 6-week treatment period showed no marked differences among groups (Supplementary Figure S1). As shown in Fig. 1B, the TS concentration was significantly increased in the serum of LoTS and HiTS-treated group of WT and NKX3.1 KO mice without any significant difference between them. But, this level was higher in HiTS-treated group of both mice than LoTS-treated group. These results confirm that serum TS concentrations were significantly increased after 6 weeks of TS injection.

### Effects of testosterone injection on the weights of testis, SV and prostate in NKX3.1 KO mice

**Fig. 2:**
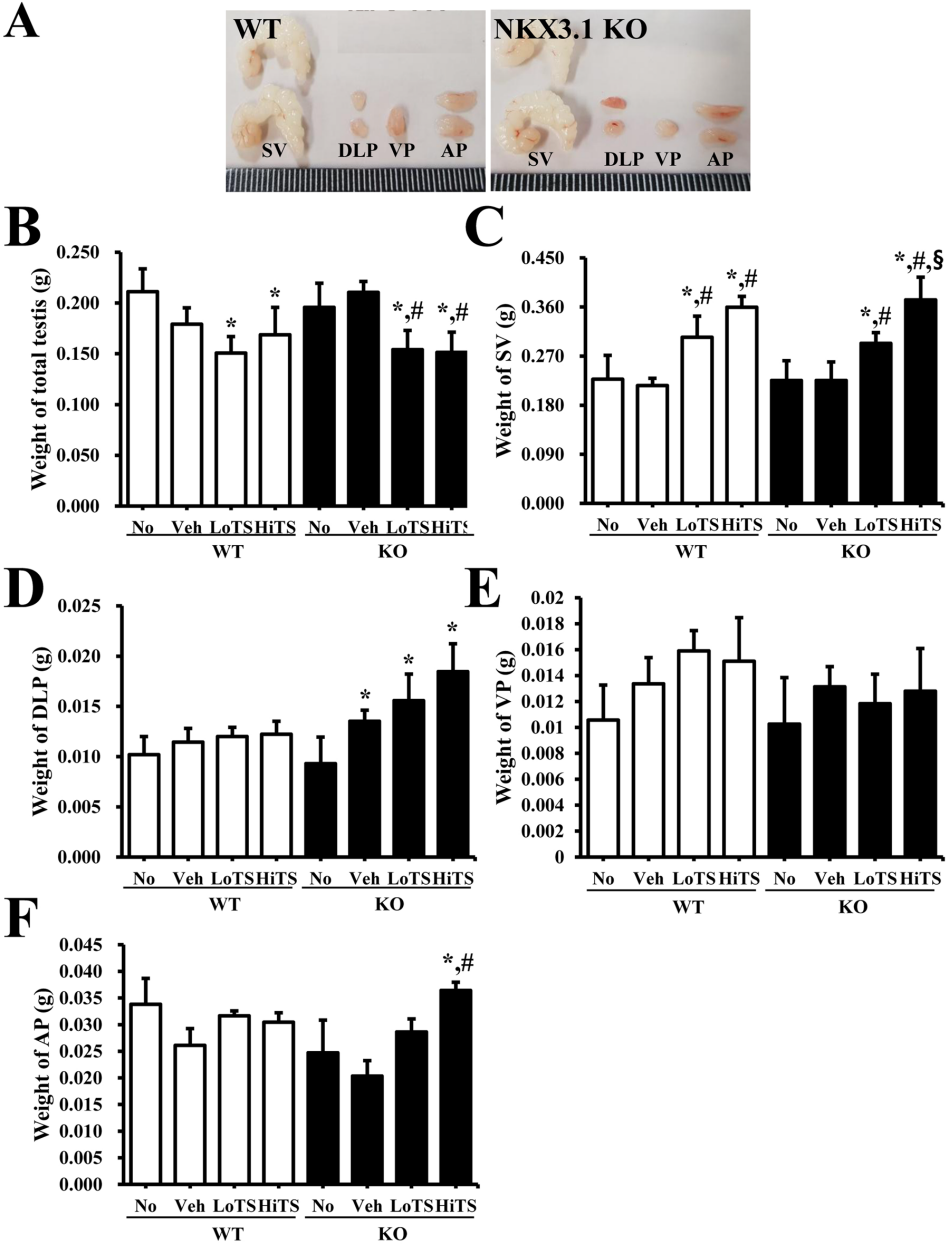
Weights of testis, SV and prostate lobes. (**A**) Actual image of male reproductive organs. (**B**) Testis weight. (**C**) SV weight. (**D**) DLP weight. (**E**) VP weight. (**F**) AP weight. Data are presented as mean ± SD. *, *p* < 0.05 vs. No; ^#^, *p* < 0.05 vs. Veh; ^§^, *p* < 0.05 vs. LoTS. Statistical significance was determined by ordinary one-way ANOVA followed by Tukey’s multiple-comparisons test. Abbreviation: wild type, WT; knockout, KO; Vehicle, Veh; low-dose testosterone, LoTS; high-dose testosterone, HiTS; seminal vesicle, SV; dorsolateral prostate, DLP; ventral prostate, VP; anterior prostate, AP

Exogenous TS is known to modulate the growth of androgen-responsive tissues, including the prostate and seminal vesicles (SV) [4, 25]. To investigated whether the injection of TS can affect the weight of male reproductive organs in NKX3.1 KO mice, alterations on the weight for testis, SV and three prostate lobes (DLP, VP and AP) were measured in TS-treated WT and NKX3.1 KO mice. The weight of testis were remarkably decreased in TS-treated group compared to Veh-treated group. But, there was no specific difference between WT and NKX3.1 KO mice (Fig. 2A and B). A reverse pattern for testis weight was detected in the weight of SV. It was higher in TS-treated group than Veh-treated group without any significant difference between WT and NKX3.1 KO mice (Fig. 2A and C). Also, the weight of DLP and AP were increased in only NKX3.1 KO mice, while WT mice was constantly remained in TS-treated groups (Fig. 2A, D and F). However, those of VP did not show a significant tendency (Fig. 2A and E). These findings demonstrate that the injection of exogenous TS may contribute to increase the weight of DLP and AP during NKX3.1 deficiency.

### Effects of testosterone injection on hyperplasia and adenoma lesions of prostate in NKX3.1 KO mice

**Fig. 3:**
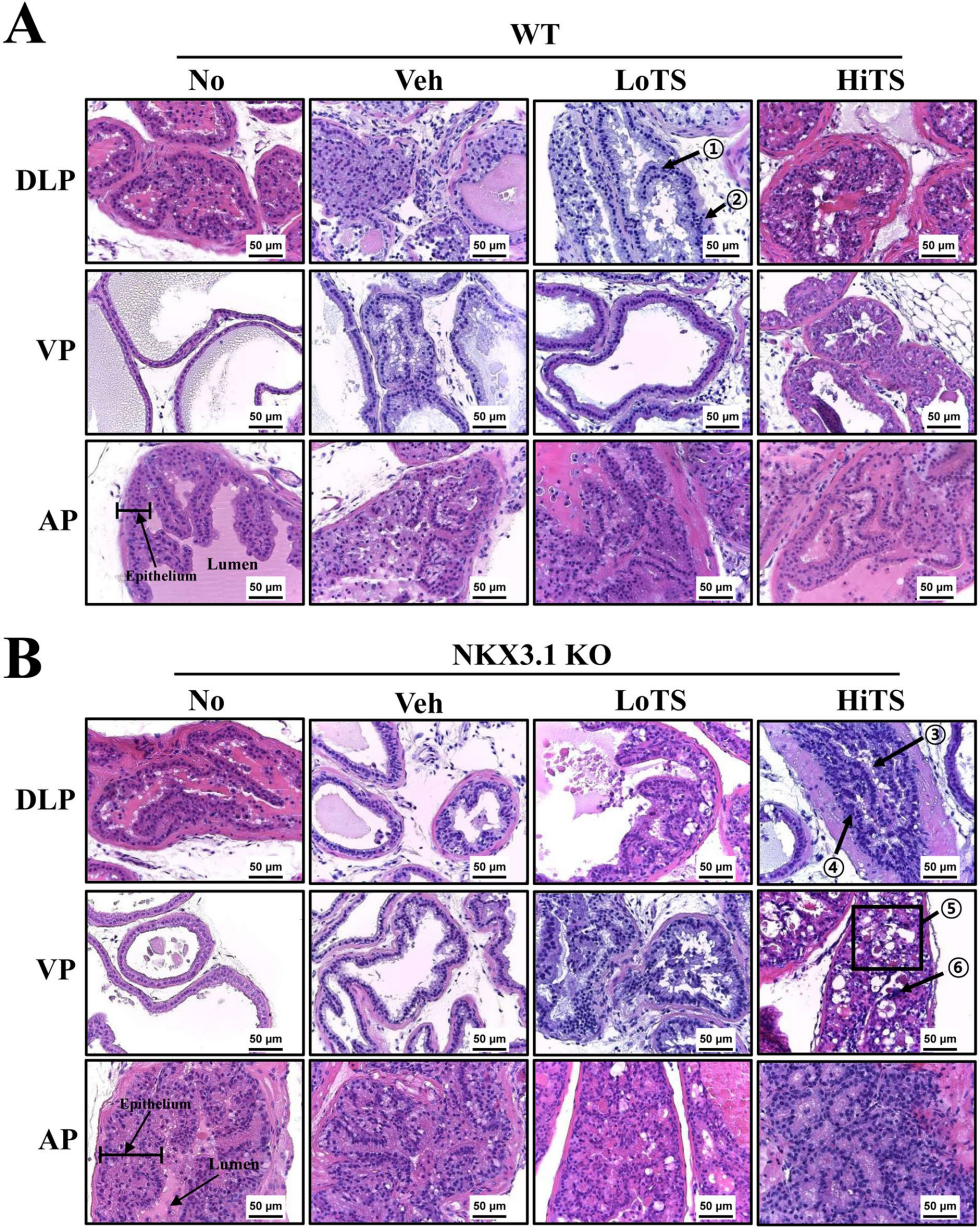
Histopathological structure of prostate lobes. (**A**) WT mice. (**B**) NKX3.1 KO mice. The section of three prostate lobes were stained with hematoxylin and eosin (H&E), and their structures were observed at 400 × magnification. Numbered arrows/box indicate representative histopathological features described in the results: 1 glandular infolding; 2 mild epithelial thickening; 3 reduced luminal space; 4 epithelial hyperplasia; 5 cribriform architecture; 6 multilayered epithelium. Abbreviation: wild type, WT; knockout, KO; Vehicle, Veh; low-dose testosterone, LoTS; high-dose testosterone, HiTS; dorsolateral prostate, DLP; ventral prostate, VP; anterior prostate, AP

**Fig. 4:**
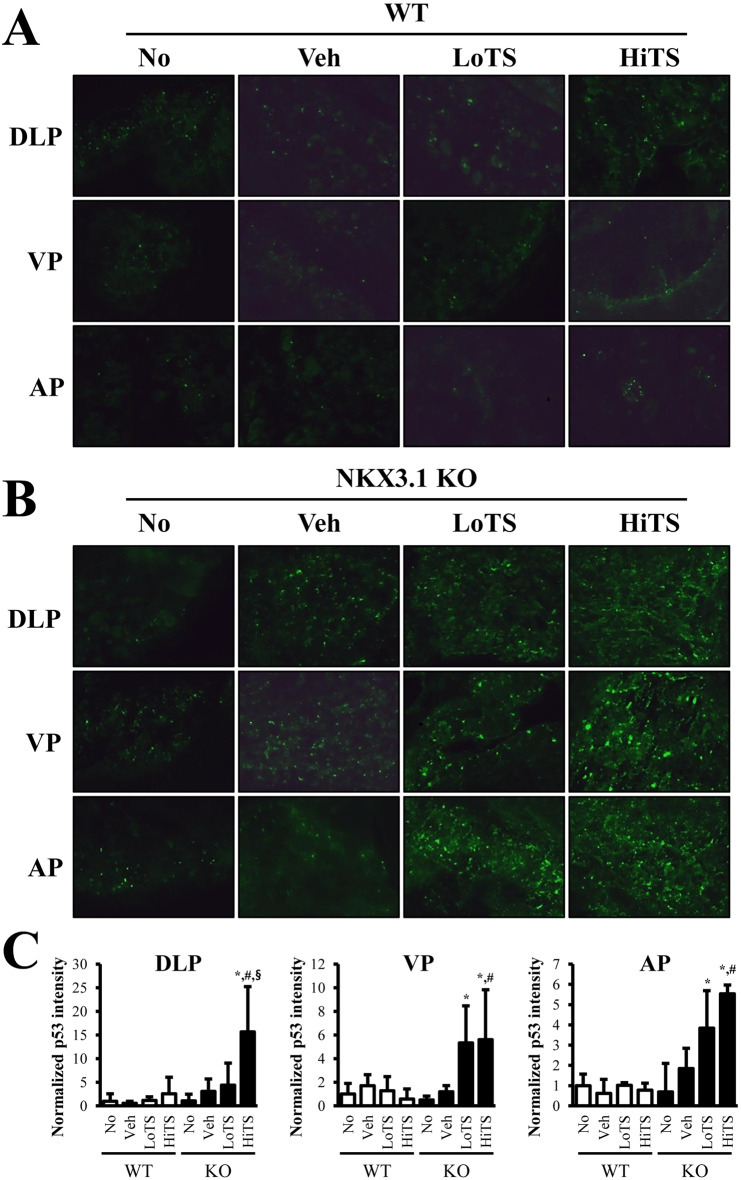
Immunofluorescence staining analysis for p53 protein. (**A**) WT mice. (**B**) NKX3.1 KO mice. The section of three prostate lobes were subjected to immunofluorescence staining using anti-p53 antibodies, and the green fluorescence signal were detected at 200 × magnification. (**C**) Quantification of p53 immunofluorescence intensity in the epithelial compartment, presented as normalized p53 intensity (fold change relative to WT). Data are presented as mean ± SD. *, *p* < 0.05 vs. No; ^#^, *p* < 0.05 vs. Veh; ^§^, *p* < 0.05 vs. LoTS. Statistical significance was determined by ordinary one-way ANOVA followed by Tukey’s multiple-comparisons test. Abbreviation: wild type, WT; knockout, KO; Vehicle, Veh; low-dose testosterone, LoTS; high-dose testosterone, HiTS; dorsolateral prostate, DLP; ventral prostate, VP; anterior prostate, AP

To investigate whether the injection of exogenous TS can induces the epithelial hyperplasia or neoplastic changes in the prostate under NKX3.1-deficient conditions, alterations on the histopathological structure of prostate were analyzed in the WT and NKX3.1 KO mice after injection of TS for 6 weeks. In WT mice, the DLP, VP, AP lobes of the No and Veh-treated groups showed the normal glandular architecture, consisting of a single layer of columnar epithelial cells and open luminal spaces (Fig. 3A). TS injection induced only mild epithelial thickening and glandular infoldings in a small subset of WT mice, with low histopathological lesion scores across the Veh-, LoTS-, and HiTS-treated groups (Table 1). In contrast, NKX3.1 KO mice exhibited the spontaneous epithelial disorganization of prostate, including irregular nuclear morphology and reduced luminal volume without TS injection (Fig. 3B). After TS injection, these histological abnormalities were markedly exacerbated. Accordingly, histopathological lesion scores tended to increase in the DLP, VP and AP of LoTS- and HiTS-treated NKX3.1 KO mice, with the highest scores observed in the HiTS-treated group (Table 1). Especially, the AP lobe of the HiTS-treated KO group showed the highest lesion score (3.4 ± 0.55) and was significantly higher than those of the Veh- and LoTS-treated WT groups, consistent with the greatest lesion severity (Table 1). These adenomatous lesions were characterized by multilayered epithelium, nuclear atypia, and cribriform glandular architecture, as shown in Fig. 3B, features indicative of early neoplastic transformation. Also, p53 immunoreactivity was increased in TS-treated NKX3.1 KO mice compared to WT mice, and this increase was more pronounced in the HiTS-treated group than in the LoTS-treated group (Fig. 4A and B), which was further supported by quantitative analysis of p53 immunofluorescence intensity (Fig. 4C). These findings demonstrate that the injection of exogenous TS exacerbates early-stage, pre-neoplastic lesions, increasing the severity of hyperplasia and adenoma in the prostate of NKX3.1 KO mice.

**Table 1:** Histopathological lesion scores in the prostates of WT and NKX3.1 KO mice after TS injection

Genotype	Groups	Lesion score		
		DLP	VP	AP
WT	Veh	0.4 ± 0.89	0.2 ± 0.45	0.4 ± 0.89
	LoTS	0.4 ± 0.89	0.4 ± 0.89	0.2 ± 0.45
	HiTS	0.4 ± 0.89	0.4 ± 0.89	0.8 ± 1.10
NKX3.1 KO	Veh	1.6 ± 1.67	0.2 ± 0.45	1.6 ± 1.67
	LoTS	1.2 ± 1.10	0.4 ± 0.55	2.0 ± 1.87
	HiTS	2.4 ± 1.34	1.8 ± 1.64	3.4 ± 0.55^*,#^

Values are presented as mean ± SD. Histopathological alterations in H&E-stained sections of the dorsolateral prostate (DLP), ventral prostate (VP), and anterior prostate (AP) were scored in a blinded manner using a 0–4 lesion severity scale: 0, normal; 1, minimal hyperplasia; 2, slight hyperplasia; 3, moderate hyperplasia; and 4, adenoma. Higher scores indicate greater lesion severity. *, *p* < 0.05 vs. WT-Veh; #, *p* < 0.05 vs. WT-LoTS, as determined by Kruskal–Wallis test followed by Dunn’s multiple-comparisons test. Abbreviations: wild type, WT; knockout, KO; Vehicle, Veh; low-dose testosterone, LoTS; high-dose testosterone, HiTS

### Effects of testosterone injection on apoptosis-related markers in the prostate of NKX3.1 KO mice

**Fig. 5:**
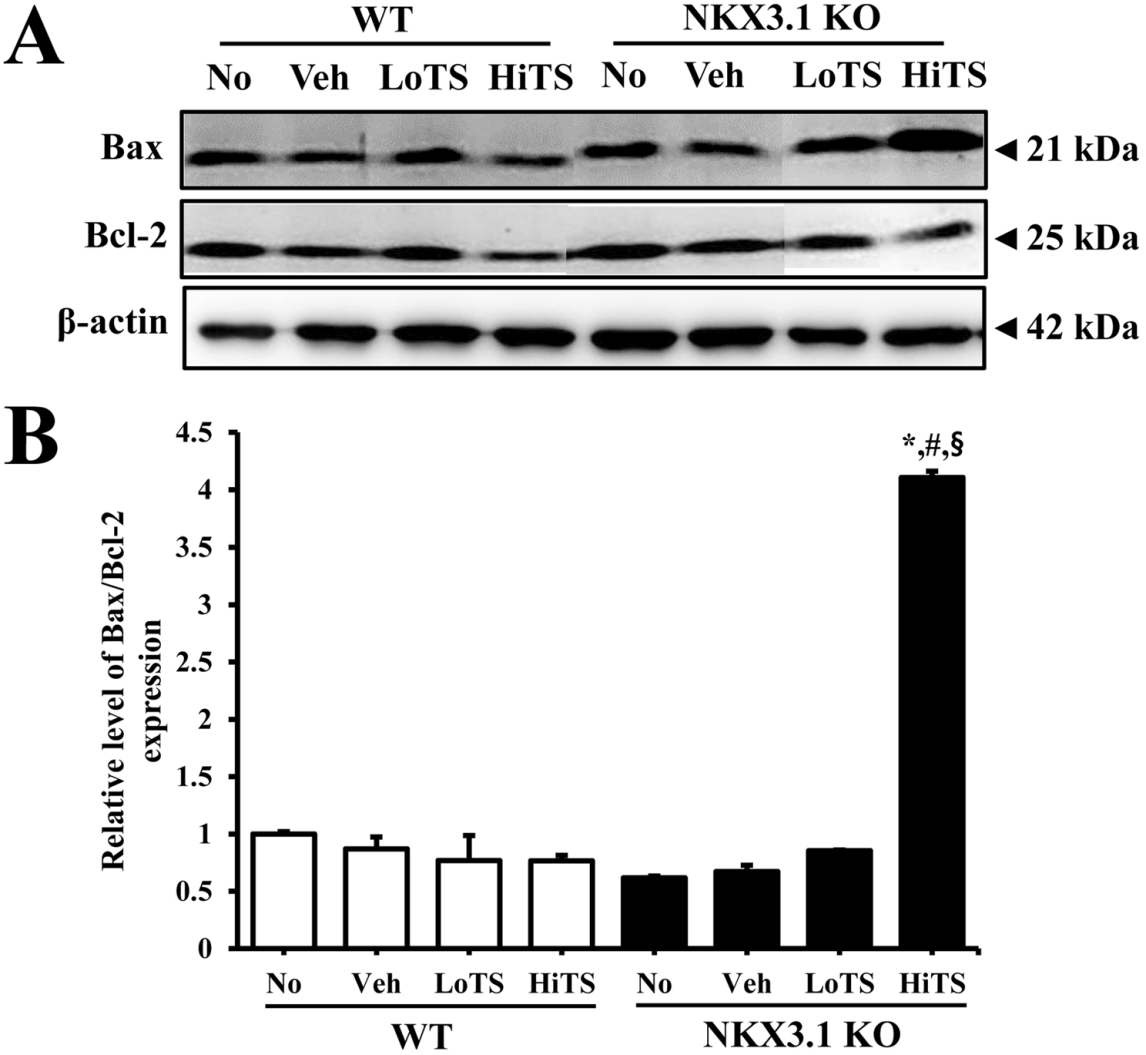
Expression levels of apoptosis-related markers in prostate. (**A**) Western blot image for Bax and Bcl-2. (**B**) Relative level of Bax and Bcl-2 protein. Each protein was detected in total prostate proteins using specific antibodies. Data are presented as mean ± SD. *, *p* < 0.05 vs. No; ^#^, *p* < 0.05 vs. Veh; ^§^, *p* < 0.05 vs. LoTS. Statistical significance was determined by Welch’s ANOVA followed by Games-Howell’s multiple-comparisons test. Abbreviation: wild type, WT; knockout, KO; vehicle, Veh; Low-dose testosterone, LoTS; high-dose testosterone , HiTS; B-cell lymphoma 2, Bcl2; bcl-2-associated X protein, Bax. Full-length uncropped blots corresponding to this figure are provided in Supplementary Figure S2

Next, we analyzed apoptosis-related markers (Bax and Bcl-2) in the prostate after TS injection. To achieve this, alterations in the expression of apoptosis-related markers, including Bax and Bcl-2, were analyzed in the prostate of NKX3.1 KO mice after injection of TS. The ratio of Bax/Bcl-2 proteins was significantly increased in only HiTS-treated NKX3.1 KO mice, while this of LoTS-treated group was remained the same as No or Veh-treated group. But, in case of WT mice, these ratios constantly remained across all groups (Fig. 5A and B). Collectively, the Bax/Bcl-2 ratio increased in the HiTS-treated NKX3.1 KO mice, whereas it remained unchanged across WT groups.

### Effects of testosterone injection on the angiogenic signaling in the prostate of NKX3.1 KO mice

As neoplastic lesion development and expansion can be accompanied by increased vascular signaling, angiogenesis is a key hallmark of early neoplastic transformation [26, 27]. Finally, we investigated whether the early-stage lesions observed in the prostate of TS-treated NKX3.1 KO mice were accompanied by activation of angiogenic signaling. To achieve this, alterations in the expression of key angiogenesis-related markers, including vascular endothelial growth factor (VEGF) and AKT (Protein kinase B, PKB), a downstream effector of VEGF signaling, were analyzed in the prostate of NKX3.1 KO mice after injection of TS.

**Fig. 6:**
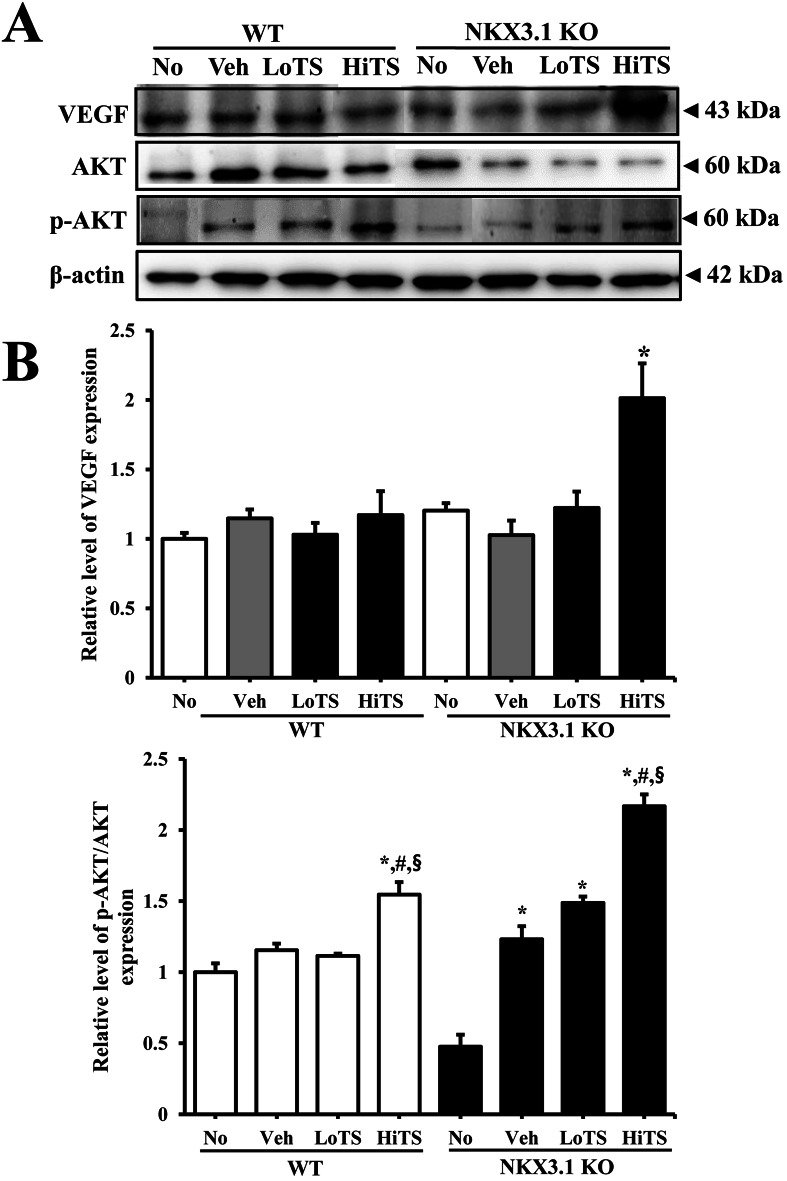
Expression levels of angiogenesis-related markers in prostate. (**A**) Western blot image for VEGF, p-AKT and AKT. (**B**) Relative level of VEGF protein and AKT phosphorylation. Each protein was detected in total prostate proteins using specific antibodies. Data are presented as mean ± SD. *, *p* < 0.05 vs. No; ^#^, *p* < 0.05 vs. Veh; ^§^, *p* < 0.05 vs. LoTS. Statistical significance was determined by Welch’s ANOVA followed by Games-Howell’s multiple-comparisons test. Abbreviation: wild type, WT; knockout, KO; vehicle, Veh; Low-dose testosterone, LoTS; high-dose testosterone, HiTS; vascular endothelial growth factor, VEGF; protein kinase B, AKT. Full-length uncropped blots corresponding to this figure are provided in Supplementary Figure S3

In WT mice, the level of VEGF expression and AKT phosphorylation did not changed neither LoTS- nor HiTS-treated group except the phosphorylation level of AKT in HiTS-treated group (Fig. 6A and B). However, in NKX3.1 KO mice, VEGF expression was increased in the HiTS-treated group relative to the No and Veh-treated groups although LoTS-treated group was remained the constant level (Fig. 6A and B). Similar increase patterns was detected in the phosphorylation level of AKT. It was remarkably higher in Veh-treated group than No group. But, this level was further increased in TS-treated group compared to Veh-treated group, the highest level of it was detected in HiTS-treated group (Fig. 6A and B). Collectively, these data indicate that the prostates of HiTS-treated NKX3.1 KO mice exhibited higher VEGF expression and increased AKT phosphorylation, consistent with an angiogenesis-associated marker profile in prostates exhibiting early-stage, pre-neoplastic lesions.

## Discussion

TS and androgen signaling have been extensively studied in prostate biology and prostate carcinogenesis [28–30]. Within this framework, the present study investigated whether exogenous TS exacerbates early-stage, pre-neoplastic lesions in the prostate of NKX3.1-deficient mice. We found that NKX3.1 KO mice showed increased susceptibility to TS exposure, with greater histopathological lesion severity, accompanied by changes in apoptosis- and angiogenesis-related markers. Together, these findings are consistent with a gene–hormone interaction in which androgenic stimulation is associated with increased severity of early-stage lesions in NKX3.1-deficient prostates.

Clinically, although our dosing paradigm does not recapitulate clinical testosterone replacement therapy (TRT), our findings may help contextualize ongoing discussions regarding TRT and prostate safety outcomes [31–33]. Given that reduced NKX3.1 expression is an early event observed in a substantial proportion of prostate cancers [8], our data suggest that NKX3.1 deficiency may modify prostate tissue responses to exogenous TS, warranting further investigation.

NKX3.1, a well-established tumor suppressor, is frequently downregulated during early-stage prostate cancer [8, 12, 34]. However, NKX3.1 deficiency alone is generally insufficient to drive invasive carcinoma without additional oncogenic stimuli, despite its contribution to tumor initiation [13, 14]. A well-characterized example is concurrent PTEN loss: NKX3.1−/−;PTEN± compound mutants develop high-grade prostatic intraepithelial neoplasia (HGPIN)/early carcinoma lesions in 60% of mice by 6 months, compared with 10% in PTEN± single mutants, whereas invasive adenocarcinoma remains rare [9]. Other cooperative contexts, including c-MYC overexpression and inflammation-associated destabilization of NKX3.1, have similarly been reported to enhance early neoplastic changes over extended time frames [35, 36]. In this context, our short-term TS exposure was associated with increased histopathological lesion severity in NKX3.1 KO mice, including adenomatous change, while WT mice exhibited only mild hyperplasia. Adenoma was observed only in NKX3.1 KO mice (25% in LoTS and 50% in HiTS), supporting that androgenic stimulation can exacerbate pre-neoplastic lesion development in an NKX3.1-deficient background.

Consistent with the observed histopathological lesion phenotypes, TS injection also increased the weight of androgen-sensitive organs, including SV, DLP, and AP, in both WT and NKX3.1 KO mice. Although both genotypes responded to TS, the increase in organ weight was more pronounced in NKX3.1 KO mice, especially following high-dose treatment, suggesting heightened androgen sensitivity in the deficient of NKX3.1. This finding is consistent with reports that NKX3.1 regulates epithelial differentiation and modulates androgen-receptor signaling [37, 38].

To place these histopathological findings in a molecular context, we assessed apoptosis-related markers in the prostate. The Bax/Bcl-2 ratio is commonly used as a descriptive readout reflecting shifts in apoptosis-related balance in tissues [39–42]. In prostate-related studies, altered expression of Bcl-2 family proteins has been reported in association with treatment responses, including androgen manipulation, and TS exposure has been shown to modulate Bax/Bcl-2 patterns in a context-dependent manner [43–45]. Consistent with this, TS has been reported to exert either anti-apoptotic or pro-apoptotic effects depending on the tissue, dose, and experimental context [46, 47]. Accordingly, Bax and Bcl-2 were included in this study as descriptive, marker-level readouts accompanying exogenous TS exposure in NKX3.1-deficient prostates. Because Bax/Bcl-2 was measured only at the terminal time point and was not accompanied by a direct assay of proliferation or apoptosis, its functional relationship to the increased lesion severity could not be determined in the present study.

We also examined angiogenesis-related signaling because vascular-associated pathways can accompany neoplastic lesion development. VEGF/AKT signaling has been implicated in vascular- and growth-associated programs in the prostate, and prior work suggests that androgen signaling and NKX3.1 status can influence VEGF-family pathways [48–55]. In our study, VEGF expression and AKT phosphorylation were elevated in TS-treated NKX3.1 KO prostates, with the highest levels in the HiTS-treated group. Together, these findings suggest that NKX3.1 deficiency may be associated with more pronounced angiogenesis-related marker responses to exogenous TS in this model.

Limitations of this study include the descriptive, marker-level nature of our molecular assessments, the absence of a validated epithelial proliferation index, the short TS exposure period, and the use of a single tumor-suppressor knockout model. An additional limitation is the relatively small group size (*n* = 5 per group), which may limit statistical power for some endpoints. Therefore, the findings should be interpreted cautiously and confirmed in larger studies. Accordingly, the observed phenotype should be interpreted primarily as early-stage, pre-neoplastic lesions (hyperplasia/adenoma) rather than as directly quantified proliferative progression. Although NKX3.1 deficiency is associated with early prostate tumorigenic changes, invasive adenocarcinoma is uncommon in this genetic context without longer observation and/or additional cooperating alterations. Future studies incorporating extended exposure durations and multi-hit models will be helpful to determine whether the gene–hormone interaction observed here influences later-stage progression.

## Conclusions

In this study, we investigated whether exogenous TS exacerbates early-stage, pre-neoplastic prostate lesions in the setting of NKX3.1-deficient mice. TS-treated NKX3.1 KO mice showed greater increases in prostate lobe weight, histopathological lesion severity, apoptosis-related marker levels, and angiogenic signaling than WT mice, with the most prominent changes observed in the HiTS-treated group. These findings highlight a synergistic interaction between androgen stimulation and NKX3.1 deficiency associated with early-stage prostate epithelial transformation. Also, our study underscores the importance of genetic context in modulating hormonal responsiveness and provides descriptive marker-level observations that accompany early prostate lesion development. Despite these insights, certain limitations should be acknowledged. The mechanistic basis of this gene–hormone synergy remains to be determined, and the relatively short experimental duration limits our ability to assess progression beyond early neoplastic changes. Furthermore, while the NKX3.1 KO model recapitulates key features of early prostate tumorigenesis, it does not fully reflect the molecular heterogeneity of human prostate cancer.

## Electronic supplementary material

Below is the link to the electronic supplementary material.

Supplementary Material 1

Supplementary Material 2

Supplementary Material 3

Supplementary Material 4

## Data Availability

All data generated or analysed during this study are included in this published article.

## Abbreviations

AKT: Protein Kinase B
AP: Anterior Prostate
DHT: Dihydrotestosterone
DLP: Dorsolateral Prostate
H&E: Hematoxylin and Eosin
HGPIN: High-grade prostatic intraepithelial neoplasia
HiTS: High-Dose Testosterone
KO: Knockout
LoTS: Low-Dose Testosterone
No: No treated
SV: Seminal Vesicles
TRT: Testosterone Replacement Therapy
TS: Testosterone
VEGF: Vascular Endothelial Growth Factor
Veh: Vehicle
VP: Ventral Prostate
WT: Wild-Type
