# Supplementary figures and images for: Exogenous testosterone exacerbates pre-neoplastic lesions in the prostate of NKX3.1-deficient mice
Source: Lab Anim Res. 2026 Jun 26;42:23. doi: 10.1186/s42826-026-00284-8 (PMC13308178; doi:10.1186/s42826-026-00284-8)

# Supplementary Figure S1

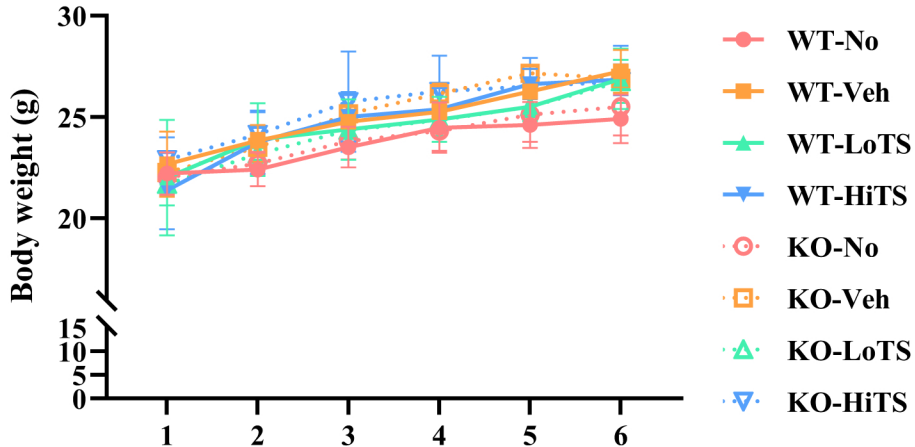

Supplement: Supplementary file 1 — Supplementary Material 1 [file 42826_2026_284_MOESM1_ESM.pdf]

# Supplementary Figure S2

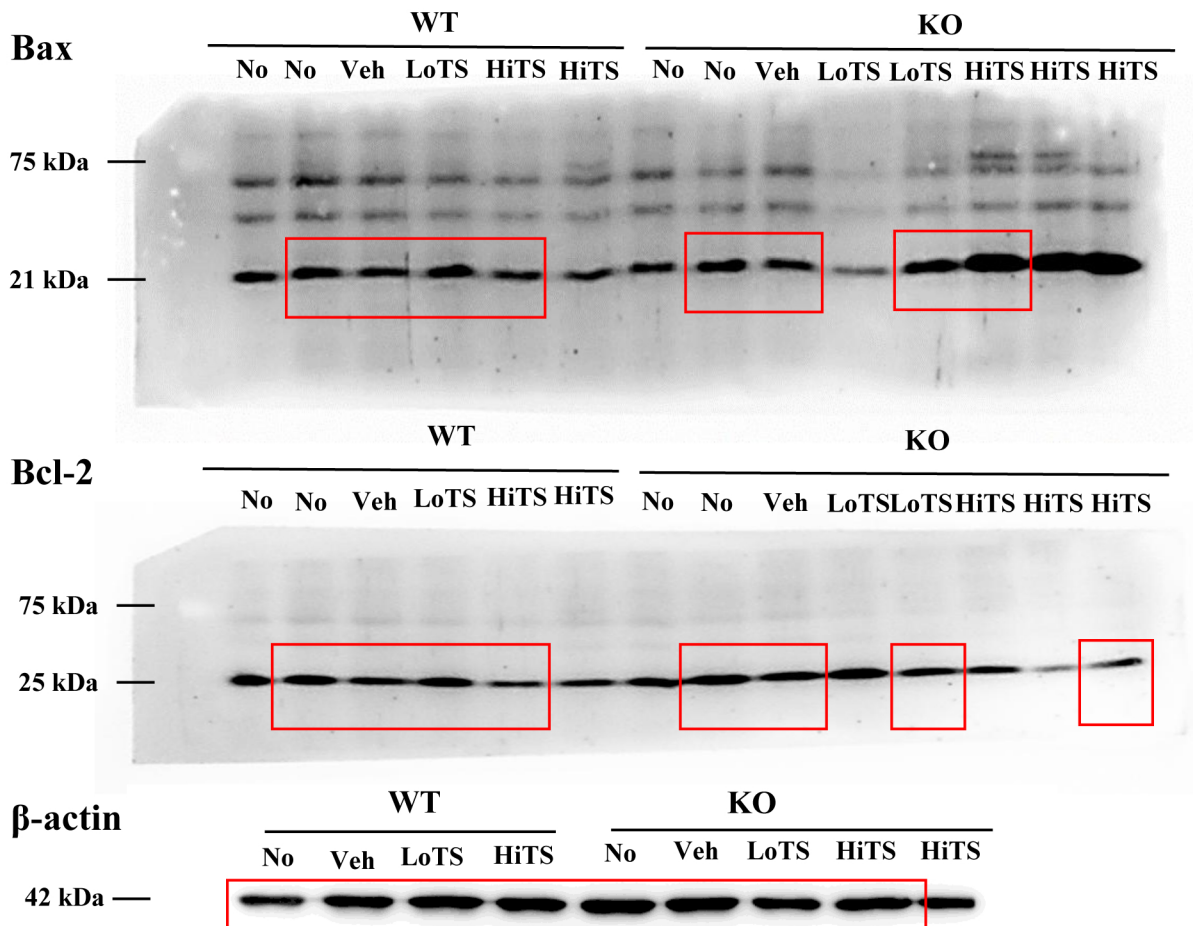

Supplement: Supplementary file 2 — Supplementary Material 2 [file 42826_2026_284_MOESM2_ESM.pdf]

Supplementary Figure S3

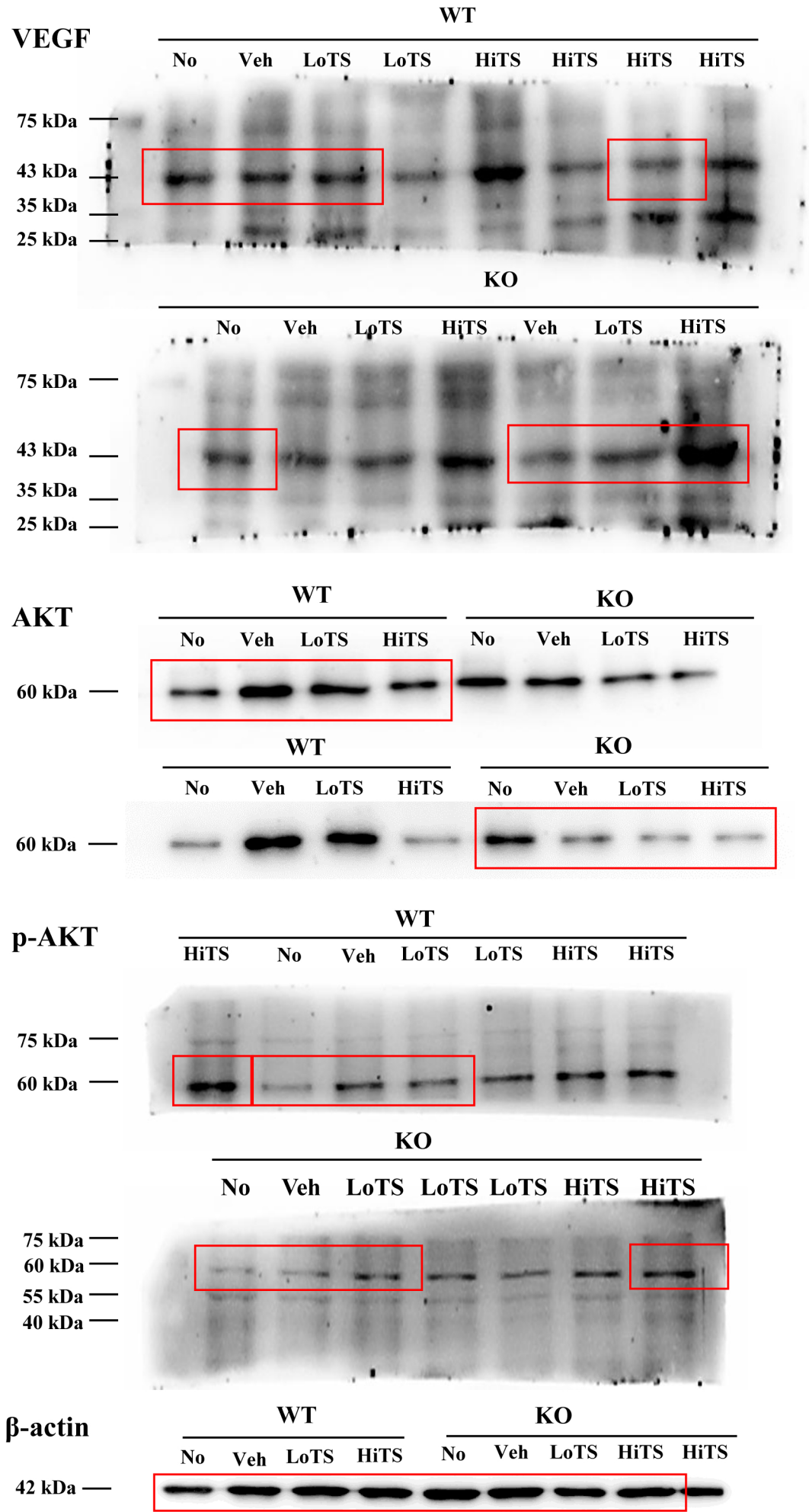

Supplement: Supplementary file 3 — Supplementary Material 3 [file 42826_2026_284_MOESM3_ESM.pdf]
