# Supplementary material for: Exogenous testosterone exacerbates pre-neoplastic lesions in the prostate of NKX3.1-deficient mice
Source: Lab Anim Res. 2026 Jun 26;42:23. doi: 10.1186/s42826-026-00284-8 (PMC13308178; doi:10.1186/s42826-026-00284-8)
Supplement: Supplementary file 4 — Supplementary Material 4 [file 42826_2026_284_MOESM4_ESM.docx]

**Supplementary Figure S1. Weekly changes in body weight in WT and NKX3.1 KO mice during 6 weeks of testosterone treatment.**

Body weight was measured weekly in WT and NKX3.1 KO mice assigned to the No, Veh, LoTS, and HiTS groups. Data are presented as mean ± SD. WT, wild-type; KO, knockout; No, non-treated; Veh, vehicle; LoTS, low-dose testosterone; HiTS, high-dose testosterone.

**Supplementary Figure S2.** Full-length uncropped blots corresponding to Figure 5. Western blot analysis of Bax (~21 kDa), Bcl-2 (~25 kDa), and β-actin (~42 kDa) in prostate tissues from WT and NKX3.1 KO mice treated with vehicle, low concentration testosterone (LoTS), or high concentration testosterone (HiTS). Red boxes indicate the cropped regions displayed in the main figure.

**Supplementary Figure S3.** Full-length uncropped blots corresponding to Figure 6. Western blot analysis of VEGF (~43 kDa), AKT (~60 kDa), phosphorylated AKT (p-AKT, ~60 kDa), and β-actin (~42 kDa) in prostate tissues from WT and NKX3.1 KO mice treated with vehicle, low concentration testosterone (LoTS), or high concentration testosterone (HiTS). Red boxes indicate the cropped regions displayed in the main figure.
